# Health-related quality of life and its socio-economic and cultural predictors among advanced cancer patients: evidence from the APPROACH cross-sectional survey in Hyderabad-India

**DOI:** 10.1186/s12904-019-0465-y

**Published:** 2019-11-05

**Authors:** Jean Jacob, Gayatri Palat, Naina Verghese, Priya Chandran, Vineela Rapelli, Sanjeeva Kumari, Chetna Malhotra, Irene Teo, Eric Finkelstein, Semra Ozdemir

**Affiliations:** 1 MNJ Institute of Oncology and Regional Cancer Center (MNJIORCC), Hyderabad, Telangana India; 2 Department of Supportive Care, Princess Margaret Cancer Centre, University Health Network, Toronto, Canada; 3 Lien Centre for Palliative Care, Duke-NUS Medical School, Singapore, 169857 Singapore; 4 Saw Swee Hock School of Public Health, National University of Singapore, Singapore, Singapore

**Keywords:** Advanced cancer, Quality of life, Well-being, India, End-of-life care, Palliative care, Palliative oncology, Socio-demographic predictors, Vulnerable

## Abstract

**Background:**

Patients with advanced cancer often experience poor health-related quality-of-life (HRQoL) due to cancer and treatment-related side-effects. With India’s palliative care landscape in its infancy, there is a concern that advanced cancer patients, especially individuals who are from disadvantaged populations experience poor HRQoL outcomes. We aim to assess HRQoL of advanced cancer patients in terms of general well-being (physical, functional, emotional, and social/family well-being), pain experiences, psychological state, and spiritual well-being, and determine the relationship between belonging to a disadvantaged group and HRQoL outcomes. We hypothesize that patients from disadvantaged or minority backgrounds, identified in this paper as financially distressed, female, lower years of education, lower social/family support, minority religions, and Non-General Castes, would be associated with worse HRQoL outcomes compared to those who are not from a disadvantaged group.

**Methods:**

We administered a cross-sectional survey to 210 advanced cancer patients in a regional cancer center in India. The questionnaire included standardized instruments for general well-being (FACT-G), pain experiences (BPI), psychological state (HADS), spiritual well-being (FACT-SP); socio-economic and demographic characteristics.

**Results:**

Participants reported significantly lower general well-being (mean ± SD) (FACT-G = 62.4 ± 10.0) and spiritual well-being (FACT-SP = 32.7 ± 5.5) compared to a reference population of cancer patients in the U.S. Patients reported mild to moderate pain severity (3.2 ± 1.8) and interference (4.0 ± 1.6), normal anxiety (5.6 ± 3.1) and borderline depressive symptoms (9.7 ± 3.3). Higher financial difficulty scores predicted most of the HRQoL domains (*p* ≤ 0.01), and being from a minority religion predicted lower physical well-being (*p* ≤ 0.05) and higher pain severity (p ≤ 0.05). Married women reported lower social/family well-being (p ≤ 0.05). Pain severity and interference were significant predictors of most HRQoL domains.

**Conclusions:**

Advanced cancer patients, especially those with lower financial well-being and belonging to minority religions, reported low physical, functional, emotional, social/family, and spiritual well-being, and borderline depressive symptoms. Future studies should be directed at developing effective interventions supporting vulnerable groups such as those with financial distress, and those belonging to minority religions.

## Background

Cancer caused 813,000 deaths in India in 2016 and accounts for above 8% of total deaths in the country [1]. Although cancer incidence in India represents only a quarter of that recorded in Europe, cancer-related death rates come close to figures in high income countries [2]. High cancer mortality in India can be attributed to low awareness about symptoms and risk factors [3, 4], high stigma of cancer in the community [5], poor access to health care [6], and high out-of-pocket costs [7, 8]. As a result, approximately 50–70% of patients present with advanced stages of cancer during their first consultation with physicians [9–11].

Patients with advanced cancer often experience poor quality of life due to cancer and treatment related side-effects [12]. To address the needs of these patients, international and local organizations have suggested that palliative care should be integrated into standard care with the goal of improving quality of life of patients [13, 14]. Health-related quality of life (HRQoL), as targeted by palliative care, is a multi-dimensional concept related to physical, functional, psychological, emotional, social, and spiritual well-being. Physical well-being, including symptom and pain management, is a priority area for oncologists and palliative care specialists alike. Equally concerning is the patients’ psycho-social well-being, associated with cancer progression and cancer-related death [15–17]. Yet, spiritual well-being has often been overlooked despite low spiritual wellbeing being associated with desire for death, hopelessness, and suicidal ideation [18–21]. Assessing HRQoL in different domains has been advocated to guide health care delivery to meet the unmet supportive care needs of advanced cancer patients [22, 23].

Although palliative care has been in existence for almost three decades in India, it is still in its infancy due to barriers such as population density, restrictive policies in opioid prescription, lack of palliative care training for medical professionals, and low awareness [24, 25]. As such, there is a concern that advanced cancer patients in India experience poor HRQoL outcomes. This is especially a concern for individuals who are from disadvantaged populations in terms of social and economic factors. Indian government identifies socially disadvantaged groups as those from Scheduled Tribes, Backward Castes and minorities such as Muslims, Christians, Sikhs, Buddhists and Zorastrians.^1^ We also recognized economically disadvantaged groups in India such as those with financial difficulties [26], lower education as well as females due to gender disparity [27], those with lower social/family support, including unmarried individuals [28] and married women who are seen as subordinates of not only men but also the older women in the household [29].

In this study, we aim to assess HRQoL in terms of general well-being (physical, functional, emotional, and social/family well-being), pain experiences, psychological state, and spiritual well-being from a cross-sectional survey with advanced cancer patients in a regional cancer center in Hyderabad, India. We expect patients in our sample to report lower HRQoL compared to cancer patients in high-income countries. Webster et al. [30] recommend interpreting the scores compared to normative data to facilitate meaningful interpretation of HRQoL in patient populations. Since these instruments were not used (or necessary scores were not provided) in an Indian population, we compared the FACT-G scores with those from cancer patients [31] and FACIT-SP scores with those from advanced cancer patients [32] in the US.

We also aim to investigate the extent to which belonging to a disadvantaged group is associated with all HRQoL outcomes. We hypothesize that patients from disadvantaged or minority backgrounds, identified in this paper as financially distressed, female, married women, lower years of education, minority religions, and Non-General Castes, would be associated with worse HRQoL outcomes compared to those who are not from a disadvantaged group. Furthermore, despite evidence that pain interferes with daily activities and affects quality of life, little is known on which specific HRQoL domains pain is associated with after adjusting for socio-demographic characteristics. In addition, culture and religion could mitigate how patients perceive pain and interference of cancer with their lives. For example, Hinduism promotes coping with pain by accepting it as a consequence of past inappropriate action (i.e. unfolding of karma) and as a means of leading to progress on a spiritual path [33]. We therefore investigate the relationships between pain and HRQoL domains in the local context, and hypothesize that pain would significantly predict physical, functional, emotional and social/family well-being, anxiety, and depression.

We expect findings from this study to provide valuable information to identify patients who experience worse HRQoL outcomes, and gaps in healthcare delivery to advanced cancer patients in a regional cancer center in India, which provides cancer treatment and palliative care. We also hope that the findings from this study will provide insights to other cancer centers in India and other developing countries.

## Methods

### Participants and setting

This study was part of a multi-country cross-sectional survey titled “Asian Patient Perspectives Regarding Oncology Awareness, Care and Health (APPROACH)” to assess gaps in care received by advanced cancer patients at major public hospitals in low- and middle-income countries (LMIC) in Asia. The study site is Mehdi Nawab Jung Institute of Oncology and Regional Cancer Center (MNJIORCC) in Hyderabad, India. It is the only government cancer hospital in Telangana and Andhra Pradesh, serving a population of 85 million, which provides cancer treatment and palliative care free of cost to all patients, sponsored by the government. The oncology department sees 12,000 new cancer patients annually and more than half the patients present with advanced cancer at the time of diagnosis.

The inclusion criteria at this site was being over the age of 21, being diagnosed with solid tumor stage 4 cancer, and being aware of diagnosis. We recruited only patients with solid cancer since patients with hematological malignancies tend to have a very different quality of life trajectory as well as prognosis compared to those with solid tumors [34]. In order to avoid having a very heterogeneous sample, we also excluded those on hospice or home-based care or those taking only palliative care treatment for more than 2 weeks. Eligible patients were recruited between September 2017 and December 2017 at inpatient wards and outpatient clinics of medical and radiation oncology departments of the MNJIORCC. The study was approved by the Institutional Ethics Committee of MNJIORCC and the National University of Singapore.

**Fig. 1:**
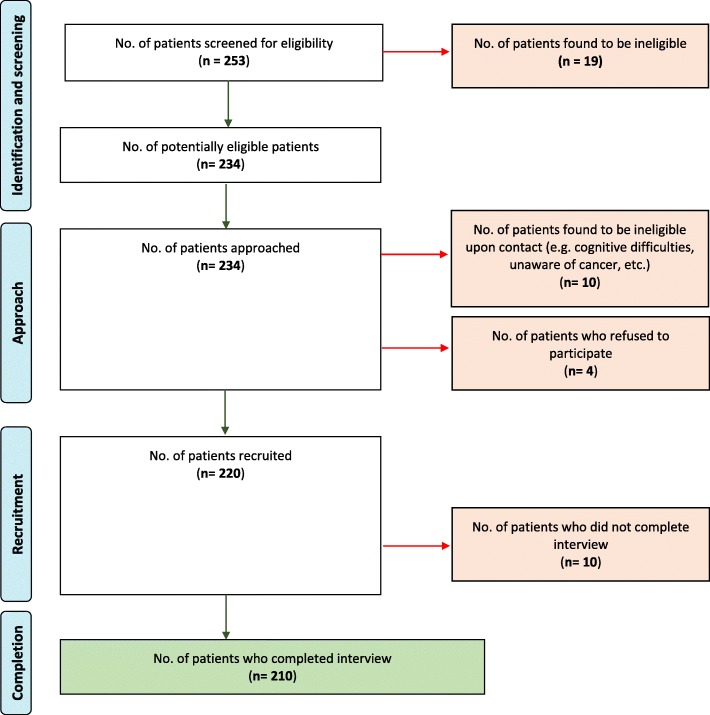
Patient recruitment log

Targeting a sample of 200 patients, the research team screened 253 medical records for the eligibility criteria and 234 patients were identified as eligible (Fig. 1). Trained interviewers then approached these patients and found that 10 deemed ineligible, either because they were not aware of their cancer or they were cognitively impaired or lacked capacity to complete the survey (which was either observed by the interviewers or informed by accompanying family caregivers of the patients). From 224 patients, 4 refused to be part of the study since they were not interested. From 220 patients who consented to the study, 10 did not complete the interview after they felt too fatigue to continue to the survey. This resulted in a response rate of 83% (100*210/234).

### Survey development

The questionnaire was developed in consultation with oncologists and comprised questions developed by the study investigators as well as validated instruments (Table 1).

**Table 1:** List of HRQoL instruments used in the study

Survey Instrument		Total Range	Sub-Domains		Sub-range
FACT-G	Functional Assessment of Cancer Therapy- General questionnaire	0 to 108	FACT-GP	Physical well-being	0 to 28
			FACT-GS	Social well-being	0 to 28
			FACT-GE	Emotional well-being	0 to 24
			FACT-GF	Functional well being	0 to 28
BPI	Brief Pain Inventory questionnaire	0 to 10	BPI-S	Pain severity	0 to 10
			BPI-I	Pain interference	0 to 10
HADS	Hospital Anxiety and Depression Scale	0 to 42	HADS-A	Anxiety	0 to 21
			HADS-D	Depression	0 to 21
FACIT -SP	Functional Assessment of Chronic Illness Therapy - Spiritual Well-being questionnaire	0 to 48	FACIT-SPMP	Spiritual meaning/ peace	0 to 32
			FACIT-SPF	Faith	0 to 16

#### HRQoL outcomes

The survey instrument included the 27-item Functional Assessment of Cancer Therapy-General questionnaire (FACT-G) to assess general well-being of patients in physical (FACT-GP), functional (FACT-GF), emotional (FACT-GE) and social/ family (FACT-GS) well-being domains after being linguistically validated in the local language [35]. We reverse scored negatively-phrased questions and summed item responses so that a high score indicated higher well being [30]. The scores for the individual domains were added to obtain a total score ranging from 0 to 108.

The pain severity (BPI-S) and pain interference (BPI-I) was assessed using the Brief Pain Inventory (BPI) questionnaire [36]. Pain severity was assessed averaging responses for four questions which assessed pain severity. Each question asked respondents to report pain on a scale of 0 (no pain) to 10 (pain as bad as you can imagine) at its worst, least, average, and at the time of responding to the questionnaire in the last 24 h. Pain interference was scored from 0 (does not interfere) to 10 (completely interferes) based on 7 questions which assessed the extent to which pain interfered with general activity, mood, walking ability, normal work, relations with people, sleep, and enjoyment of life.

The questionnaire also included the Hospital Anxiety and Depression Scale (HADS) measuring anxiety (HADS-A) and depression (HADS-D) domains with 7 questions each [37]. We reverse scored positively-phrased statements and summed item responses so that higher score indicated higher psychological distress. Possible scores ranged from 0 to 21 for anxiety and 0 to 21 for depression and the resulting total score ranged from 0 to 42. A score of 0 to 7 for either subscale is regarded as the normal range, a score of 8 to 10 is suggestive of the presence of the respective state, and a score of 11 or higher suggests probable presence of the mood disorder [38].

Spiritual well-being was assessed using the Functional Assessment of Chronic Illness Therapy-Spiritual Well-being questionnaire (FACIT-SP) consisting of two domains: spiritual meaning/peace (FACT-SPMP), and faith (FACIT-SPF) [39]. Items in the first domain emphasize meaning, harmony, and peacefulness, where items in the second domain focus on a sense of strength and comfort from one’s spiritual beliefs. A similar scoring system to FACT-G was used. The resulting total spiritual well-being score ranged from 0 to 48.

For all scales, we dropped respondents from scoring if they did not answer at least 50% of the items in a scale. If they answered more than 50% but skipped some of the items, we prorated the scores using the average of the answers of the other respondents. Table 1 presents information on the sub-domains and scores. These scales were chosen by the study investigators as they have been commonly used in multiple countries for cancer patients [40–42].

#### Socio-demographic characteristics

Financial difficulty was measured by summing responses across three questions: (i) How well does the amount of money you have enable you to cover the cost of your treatment?; (ii) How well does the amount of money you have take care of your daily needs?; and (iii) How well does the amount of money you have enable you to buy those little ‘extras’, that is, those small luxuries?. The last two questions were taken from the economic well-being section of the Older American Resources and Services survey [43], and the first was added by the study investigators. Responses were coded as 1 for ‘Very well’, 2 for ‘Fairly well’, and 3 for ‘Poorly’. The resulting score ranged from 3 to 9, where 3 represented the lowest financial difficulty and 9 indicated the highest.

The survey instrument also included questions on demographic characteristics, including age, years of education, marital status, religion, and caste category.

#### Translation

The questions designed by the study investigators were first developed in English (Additional file 1 presents the English questionnaire). Subsequently, they were translated by professional translators into Telugu, which is spoken by the majority of the population at the study site, and then back translated into English. The original and back translated English versions were compared and reconciliations were made where necessary. Further revisions were made to these questions based on feedback from the physicians and cognitive interviews with 10 eligible patients in the study site.

Licensed Telugu translations for BPI and HADS were obtained from license owners of survey instruments. FACT-G and FACIT-SP instruments were translated according to a strict translation protocol laid out by its license owners, involving two forward and back translations, and a reconciliation and testing of the instruments in the translated language. The instruments were tested with 10 patients. After patients went through the main survey instrument (without the FACT-G and FACIT-SP instruments), they were asked to complete the FACT-G and FACIT-SP and were asked to complete a briefing questionnaire investigating whether patients understood and interpreted the questions correctly. The instruments were then finalized after discussions among the license owner, study investigators and translators. The final translation was approved by the FACIT license owner.

### Statistical analysis

Internal consistency of FACT-G, BPI and FACIT-SP and HADS were measured by Cronbach alpha [44]. To compare the FACT-G and FACIT-SP scores with those from U.S-based studies, we calculated Cohen’s D effect sizes to compare the means from the two samples, and effect sizes 0.8 and over were considered to be large [45].

We used ordinary least square (OLS) regressions to investigate the association between the HRQoL outcomes and predictors. A separate model was estimated for each HRQoL outcome, where the dependent variable was patients’ self-reported quality of life (FACT-G, FACT-GP, FACT-GF, FACT-GE, FACT-GS), pain experiences (BPI-S, BPI-I), anxiety and depression (HADS, HADS-A, HADS-D), and spiritual well-being (FACIT-SP, FACT-SPMP, FACIT-SPF), totaling 16 models. The predictors or independent variables were financial difficulties score, gender (female = 1, male = 0), years of education, marital status (unmarried (separated/divorced/widowed/never married) = 1, otherwise = 0), religion (non-Hindu = 1, Hindu = 0), and type of caste (non-General Caste = 1, General Caste = 0). Age was included in all regression as a control variable.

We used marital status as a proxy for social/family support in the analysis, where being married represented higher social/family support. However, married women from disadvantaged backgrounds have a low status within the household especially if they live with their in-laws [29]. We thus investigated whether the effect of marital status on social/family well-being varies based on gender by adding an interaction effect between marital status and gender.

To examine association of BPI-S and BPI-I with HRQoL domains, we also used OLS models adjusting for covariates (financial difficulties, gender, education, marital status, religion, caste and age) described above. Statistical significance was measured at the 5% level. All analyses were conducted at STATA 14.

## Results

### Patient characteristics

Table 2 presents the demographic information of the respondents. The mean age of the patients was 49 years and mean years of education was 2.8 years. Approximately 52% (95% confidence interval (CI):46–59%) of the patients were female. Most patients reported that they belonged to disadvantaged castes or tribes (84%; CI: 79–89%), were Hindus (83%; CI: 78–88%) and married (75%; CI: 69–81%). The most common cancer types were lung (23%; CI: 18–29%), breast (23%; CI: 17–29%), cervical (15%; CI: 10–20%) and oral (11%; CI: 7–15%) cancer. Most patients (88%; CI: 83–92%) were recruited from inpatient clinics. The mean financial difficulties score was 7.9 (Standard Deviation (SD): 1.4) (3 reflecting the least and 9, the most financial difficulty).

**Table 2:** Patient Characteristics (*N* = 210)

Characteristics	Statistics
Age in years, mean (S.D.), range	49.1 (11.9), 20–84
Years of education, mean(S.D.), range	2.8 (4.5), 0–18
Gender, N (%)	
Male	100 (47.6%)
Female	110 (52.4%)
Marital status, N (%)	
Married	158 (75.2%)
Unmarried	52 (24.8%)
Religion, N (%)	
Hindu	174 (82.9%)
Others	36 (17.1%)
Caste, N (%)	
General	34 (16.2%)
Non-general Caste (Scheduled Caste, Scheduled Tribe, Other Backward Class, Don’t know)	176 (83.8%)
Financial difficulty score ^a^, mean (S.D.), range	7.9 (1.4), 3–9
Patient type, N (%)	
Outpatient	26 (12.4%)
Inpatient	184 (87.6%)
Type of cancer, N (%)	
Lung	49 (23.3%)
Breast	48 (22.9%)
Cervical	32 (15.2%)
Oral	23 (11%)
Colorectal	6 (2.9%)
Gastric	14 (6.7%)
Head and Neck	10 (4.8%)
Other	28 (13.3%)

^a^Financial difficulties score ranges from 3 to 9, where 3 is the lowest financial difficulty and 9 is the highest financial difficulty score

### Internal consistency reliability

Except for the faith subscale score (0.48), the total scores and sub-scales scores from FACT-G (total score = 0.79, physical well-being score = 0.64, social/family well-being score = 0.66, emotional well-being score = 0.72 and functional well-being score = 0.71), BPI (total score = 0.89, pain severity score = 0.88 and pain interference score = 0.90), HADS (total score = 0.79, anxiety score = 0.77 and depression score = 0.65), and FACIT-SP (total score = 0.80, meaning score = 0.83) had good internal consistency reliability.

### Findings on HRQoL outcomes

Patients in our sample reported a mean score of 62 (SD: 10) for FACT-G (Table 3). Compared to the U. S patients, the reported general well-being scores were lower in all subscales, especially for functional well-being (effect size (ES) = 1.5; CI: 1.6–1.3) and social/ family well-being (ES = 1.1; CI: 1.3–1.0), indicating worse HRQoL than the U. S sample. The mean pain severity and pain interference scores were 3.2 (SD: 1.8) and 4 (SD: 1.6) out of 10, respectively, signifying mild to moderate pain. The mean scores for the HADS anxiety and depression scores were 5.6 (SD: 3.1) and 9.7 (SD: 3.3), respectively, indicating normal anxiety but suggestive of borderline depressive symptoms for the patients in our sample. Patients also reported a mean score of 33 (SD: 5.5) for spiritual well-being which is worse than that of the advanced cancer patients in the U. S (ES = 1.1; CI: 1.3–0.9).

**Table 3:** Patient-reported HRQoL outcomes

Instrument	Hyderabad Patients (*N* = 210)	U.S. cancer sample (*N* = 2236)^a^ (*N* = 156)^b^	Cohen’s D
	*Mean (S.D.)*	*Mean (S.D.)*	*Mean (95% CI)*
General well-being			
FACT-G total score (0–108)	62.4 (10.0)	80.9 (17.0)	−1.1 (− 1.3, − 1.0)
FACT-GP: Physical well-being sub-scale (0–28)	17.0 (4.5)	21.3 (6.0)	− 0.7 (− 0.9, − 0.6)
FACT-GS: Social/ Family well-being sub-scale (0–28)	16.2 (3.3)	22.1 (5.3)	− 1.1 (− 1.3, − 1.0)
FACT-GE: Emotional well-being sub-scale (0–24)	20.0 (3.7)	18.7 (4.5)	− 0.29 (0.2, 0.4)
FACT-GF: Functional well-being sub-scale (0–28)	9.2 (3.8)	18.9 (6.8)	− 1.5 (− 1.6, − 1.3)
Pain experiences			
BPI-S: Pain severity (0–10)	3.2 (1.8)		
BPI-I: Pain interference (0–10)	4 (1.6)		
Anxiety/ depression			
HADS total score (0–42)^c^	15.3 (5.6)		
HADS-A: Anxiety sub-scale (0–21)	5.6 (3.1)		
HADS-D: Depression sub-scale (0–21)	9.7 (3.3)		
Spiritual well-being			
FACIT SP total score (0–48)	32.7 (5.5)	39.7 (7.2)	−1.1 (−1.3, −0.9)
FACIT-SPMP: Meaning/ Peace sub-scale (0–32)	20.1 (4.3)		
FACIT-SPF: Faith sub-scale (0–16)	12.5 (2.7)		

^a^FACT-G is referenced from Brucker PS, Yost K, Cashy J, Webster K, Cella D. General Population and Cancer Patient Norms for the Functional Assessment of Cancer Therapy-General (FACT-G). Evaluation & the Health Professions. 2005;28(2):192–211

^b^FACIT SP is referenced from Daugherty, C. K., Fitchett, G., Murphy, P. E., Peterman, A. H., Banik, D. M., Hlubocky, F., & Tartaro, J. (2005). Trusting God and medicine: Spirituality in advanced cancer patients volunteering for clinical trials of experimental agents. Psycho-Oncology, 14(2), 135–146

^c^ HADS sub-scales: Normal = 0–7; Borderline abnormal = 8–10; Abnormal = 11–2

### Predictors of HRQoL outcomes

Table 4 presents outcomes of the OLS regressions. As hypothesized, patients with higher financial difficulty scores reported lower functional well-being (FACT-GF), lower emotional well-being (FACT-GE), lower meaning/peace subscale of the spiritual well-being (FACIT-SPMP), and higher anxiety and depressive symptoms (HADS, HADS-A, HADS-D). Also consistent with our hypothesis, non-Hindu patients reported lower physical well-being (FACT-GP) and higher pain severity compared to Hindu patients.

**Table 4:** Linear regressions of HRQoL outcomes on patient characteristics

	1	2	3	4	5	6	7	8	9	10	11	12	13
Variables	FACT-G	FACT-GP	FACT-GF	FACT-GE	FACT-GS	BPI-S	BPI-I	HADS	HADS-A	HADS-D	FACIT-SP	FACIT-SPMP	FACIT-SPF
Financial difficulty score (3–9)	− 0.641	0.311	0.438***	0.627***	0.113	0.0831	0.109	1.185***	0.396***	0.789***	−1.312***	− 1.238***	− 0.0738
	(− 0.435)	(− 0.205)	(− 0.163)	(− 0.162)	(− 0.159)	−(0.0881)	(− 0.0781)	(−0.245)	(− 0.137)	(−0.142)	(− 0.256)	(−0.196)	(− 0.127)
Female	−2.018	−1.228	−0.802	− 0.125	0.137	0.129	0.0395	−0.0242	0.287	−0.312	0.272	−0.195	0.467
	−1.581	−0.648	−0.587	− 0.566	−0.52	− 0.29	−0.268	− 0.844	−0.459	− 0.479	−0.824	− 0.582	−0.425
Years of education	−0.127	0.0343	−0.0633	−0.0404	− 0.0577	−0.0107	− 0.0129	0.0209	0.0676	−0.0466	− 0.0745	−0.0845	0.0101
	−0.186	−0.0827	− 0.0658	−0.0645	− 0.0572	−0.0326	− 0.0325	−0.0835	− 0.0501	−0.0449	− 0.103	−0.0676	− 0.0552
Unmarried	0.473	0.0617	−0.0274	−0.86	1.299**	−0.18	− 0.268	− 1.011	−0.332	− 0.679	−1.492	− 1.267	−0.225
	−1.808	−0.728	−0.649	− 0.669	−0.604	− 0.319	−0.27	− 1.029	−0.585	− 0.54	−0.886	− 0.651	−0.43
Non-Hindu	−4.522	−2.050**	−0.58	−0.68	−1.212	0.891**	0.447	1.628	1.246	0.382	1.545	0.461	1.084
	−2.342	−0.996	−0.861	−1.124	−0.797	− 0.381	−0.413	− 1.235	− 0.782	−0.7	− 1.629	−1.155	−0.814
Non-General caste	−3.546	−1.554	−0.15	−0.479	−1.363	0.745	0.383	0.479	0.643	−0.164	1.784	0.804	0.98
	−2.345	−1.058	−0.933	−0.997	− 0.816	−0.424	− 0.461	− 1.296	−0.883	− 0.692	−1.626	− 1.145	−0.89
Age	0.0192	0.038	−0.0238	0.022	−0.0171	−0.0168	− 0.00566	0.0427	0.0179	0.0248	−0.0126	−0.018	0.00536
	−0.0663	−0.028	− 0.0238	−0.0234	− 0.0227	−0.0122	− 0.0115	−0.0371	− 0.02	−0.0213	− 0.0342	−0.0266	− 0.0144
Constant	71.54***	14.85***	14.62***	24.81***	17.26***	2.597**	3.062***	3.334	0.565	2.769	42.34***	30.71***	11.63***
	−5.774	−2.445	−2.132	− 2.423	−2.11	−1.101	−1.022	−3.319	−1.801	− 1.939	−3.721	−2.694	− 1.746
Observations	210	210	210	210	210	210	186	210	210	210	210	210	210

Contrary to our hypothesis, unmarried patients reported higher social/family well-being compared to those who are married. In the expanded model with an interaction effect between gender and marital status, marital status and the interaction effect of marital status and gender were not significant predictors of social/family well-being. However, an unadjusted comparison of mean social/family well-being score via t-tests showed that married women in our sample reported lower social/family well-being than unmarried women (score for married women = 15.84; score for unmarried women = 17.79; *p* value = 0.0077). Years of education, gender, and caste had no significant association with any outcomes.

### Association between pain and other HRQoL outcomes

Table 5 presents results of 16 regressions showing whether BPI-S/ BPI-I are significant predictors of general well-being domains (FACT-G, FACT-GP, FACT-GF, FACT-GE, FACT-GS), anxiety, and depression (HADS), after adjusting for covariates. Consistent with our hypotheses, higher pain severity and higher pain interference were significantly associated with all but one (*P* > 0.10 between BPI-S and FACT-GE) domains of general well-being, anxiety, and depression (*P* ≤ 0.01 for all; but *P* ≤ 0.05 between BPI-I and FACT-GS).

**Table 5:** Association between HRQoL outcomes and pain experience

	(1)	(2)	(3)	(4)	(5)	(6)	(7)	(8)
Variables	FACT-G	FACT-GP	FACT-GF	FACT-GE	FACT-GS	HADS	HADS-A	HADS-D
BPI-S	−2.545***	−1.322***	−0.632***	−0.236	−0.355***	0.709***	0.380***	0.329***
	(0.331)	(0.137)	(0.138)	(0.122)	(0.131)	(0.205)	(0.114)	(0.116)
BPI-I	−3.596***	−1.354***	−0.986***	−0.984***	−0.272**	1.297***	0.679***	0.618***
	(0.361)	(0.160)	(0.175)	(0.175)	(0.136)	(0.198)	(0.122)	(0.114)
Observations	210	210	210	210	210	210	210	210

Coefficients (Robust standard errors)

BPI-S: 0–10 where 10 is highest pain severity; BPI-I: 0–10 where 10 is highest pain interference; HADS-A and HADS-D: 0 to 21 where 21 is the highest anxiety/ depression score

Each cell represents the beta coefficient for BPI-S/BPI-I, adjusted for financial difficulty, female, years of education, unmarried, non-Hindu, non-General caste, and age

*** *p* < 0.01, ** *p* < 0.05

## Discussion

This study used data from a cross-sectional survey of advanced cancer patients at a regional cancer center in India to primarily assess HRQoL and its socio-demographic predictors. Findings indicate that advanced cancer patients in the study setting reported lower general well-being and spiritual well-being compared to a reference population of cancer patients in the U.S. Patients reported mild to moderate pain, and responses were suggestive of normal anxiety levels but borderline depression. Higher financial difficulty scores predicted most of the HRQoL domains, and being from a minority religion predicted lower physical well-being and higher pain severity. Years of education, gender and caste were not significant predictors for patients in our sample. Married women reported lower social/family well-being. We also found that pain severity and pain interference were significant predictors of HRQoL.

### General well-being

Compared to those in the U.S. patient sample, patients in our sample reported lower functional, physical, emotional, and social/family well-being. The difference was especially striking for functional and social/family well-being. Low literacy levels (average years of education = 2.8) in our sample also suggest that patients’ and their families may not fully comprehend the disease, its affects, and how to support the patient, pushing down social/family well-being scores at the site. Furthermore, stigma associated with advanced cancer in the Indian context could further impact social/family well-being negatively. On the supply-side, the difference in well-being scores could be explained by the availability of well-resourced allied health services such as physiotherapy and social work in high income countries and the relative lack of these services in the study setting and the Indian public health system context in general [46].

### Pain severity and interference

Despite the presence of a palliative care service at the study site which prescribes opioid analgesics for management of severe cancer pain, most patients in our sample reported mild to moderate pain severity and interference. Less than 3% of cancer patients in India have access to pain relief, largely due to cumbersome processes and legislation for procuring and dispensing oral morphine in India [24]. We therefore believe that the experience of other advanced cancer patients in other parts of the country with little or no access to palliative care services may be worse than the patients in our sample.

We also found that pain interference was a predictor of HRQoL, with the largest effect on physical well-being and anxiety and depression, and pain severity a predictor of all but emotional well-being. The significant relationship between pain interference and emotional and social/family well-being also shows that pain affects patients’ lives in several dimensions. Although these results are not unique to this study [47, 48], they underscore the need for healthcare providers to give further attention to effective pain management and how pain interacts with different domains of patients’ well-being.

### Anxiety, depression and spiritual well-being

Patients in our sample also reported depressive symptom levels suggestive of borderline depression, and lower spiritual well-being than those in the U.S. These findings suggest that advanced cancer patients receive little psycho-social and spiritual support from the medical system or religious community, consistent with evidence in low and middle income countries alike [49].

### Predictors of HRQoL

Financial difficulty was the most important predictor of lower HRQoL at the site. This is consistent with findings of other studies which show that lower socio-economic status is positively associated with lower physical, psychological and social well-being [26, 50–52]. Our findings show that even in a setting where cancer treatment is free, those with higher financial difficulty scores report lower quality of life outcomes. This could be indicative of severe financial distress due to loss of daily income of patients and working caregivers [53], which could increase the burden on meeting other household expenditure needs. We recommend that even in settings where treatment is free, patients should be systematically screened for financial distress and targeted for financial support interventions.

Belonging to a minority religion was associated with lower physical well-being and higher pain severity. This could be an indicator of poorer health among those belonging to minority religions [54], which are considered socially disadvantaged by the Indian government [55]. Furthermore, we can draw comparisons from the U.S. setting, where studies examining unrelieved pain among racial minorities points towards factors such as limited access to health care and appropriate analgesics, and limited access and utilization of pain specialists [56]. In addition, this may be due to Hindu patients’ accepting pain better than those from other religions [33]. To the best of our knowledge, there are no studies comparing how those practising Hinduism perceive pain as compared to those practising other religions. However, studies show that greater acceptance of pain was associated with lower reports of pain and higher functioning [57]. Future studies could examine disparities in health, access to healthcare services, and pain management among religious groups with advanced cancer such that a greater understanding can confirm or reject our hypotheses and reveal whether or not specific interventions are warranted.

In this study, a particularly interesting result was the association between marital status and social/family support. Contrary to previous studies [50] and our hypothesis, we found marriage, a proxy for social/family support, to be a complicated indicator affected by both marital status and gender at our site. Unmarried women reported higher mean scores for social/family well-being compared to married women. Married women in India who typically have low status may lose any social support they used to have once they fall ill [58, 59]. Further studies could be explored in the Indian context to examine the impact of an advanced cancer diagnosis on the marital and family relationship and the effectiveness of psycho-social interventions to support relationships through the clinical trajectory.

### Limitations

This study has several limitations. First, patients are sampled from a single hospital in Hyderabad. As such, our results may not be nationally representative. However, the study site is the only government cancer hospital in Telangana and Andhra Pradesh, serving a population of 85 million, a substantial segment of the nation. Second, the sample was not drawn at random and we excluded those who were not aware of their cancer or were cognitively impaired to complete the survey. Unlike the western societies where patient autonomy is the norm, patients in India may not know their cancer diagnosis since families ask physicians not to disclose diagnosis in desire to ‘protect the patient’ and physicians usually comply with this request. Patients who are unaware of their cancer can constitute half of the patients [60, 61]. Hence, our sample may not be representative of the overall advanced cancer population seen in the study site or in the region. Third, the comparison of scores with the reference cancer population in the U.S. did not control for demographics and disease severity differences. However, this is the first to study the relationship between socio-demographic factors and all domains of HRQoL for advanced cancer patients in India using standardised and widely used instruments. Fourth, concerns about social stigma associated with cancer, which are quite common in India [62], may have led patients to provide socially desirable answers.

## Conclusions

We investigated HRQoL outcomes of stage 4 cancer patients in a regional hospital in India and their predictors. Advanced cancer patients in our sample reported low physical, functional, emotional, social/family, and spiritual well-being, and experienced borderline depression. This was especially the case for those with lower financial well-being and belonging to minority religions. Married women also reported lower social/family well-being. Additionally, pain interference and pain severity were associated with almost all domains of HRQoL. Future research should be directed at improving HRQoL of advanced cancer patients, and especially those as identified by our study as most vulnerable, including those with financial distress, minority religions, and married women. Future research should also assess the HRQoL experience of patients who are not aware of their cancer diagnosis or cognitively impaired.

## Supplementary information

**Additional file 1.** Survey instrument.

## Data Availability

Data is available on request.

## Abbreviations

APPROACH: Asian Patient Perspectives Regarding Oncology Awareness, Care and Health
BPI: Brief Pain Inventory
BPI-I: Pain interference
BPI-S: Pain severity
CI: Confidence Interval
EWB: Emotional well-being
FACIT- SP MP: Spiritual meaning/peace
FACIT-SP F: Faith
FACIT-SP: Functional Assessment of Chronic Illness Therapy-Spiritual Well-being
FACT-G: Functional Assessment of Cancer Therapy-General
FWB: Functional well-being
HADS: Hospital Anxiety and Depression Scale
HADS-A: Anxiety
HADS-D: Depression
HRQoL: Health-related quality of life
LMIC: Low- and middle-income countries
MNJIORCC: MNJ Institute of Oncology and Regional Cancer Center
PWB: Physical well-being
SD: Standard Deviation
SFWB: Social/ family well-being

## Footnotes

1 Planning Commission. Human and Social Development. Empowerment of the Socially Disadvantaged Groups. Accessed in June 2019. http://planningcommission.nic.in/plans/planrel/fiveyr/9th/vol2/v2c3-9.htm
