# Correction to: Health-related quality of life and its socio-economic and cultural predictors among advanced cancer patients: evidence from the APPROACH cross-sectional survey in Hyderabad-India

**DOI:** 10.1186/s12904-020-0519-1

**Published:** 2020-01-23

**Authors:** Jean Jacob, Gayatri Palat, Naina Verghese, Priya Kumari, Vineela Rapelli, Sanjeeva Kumari, Chetna Malhotra, Irene Teo, Eric Finkelstein, Semra Ozdemir

**Affiliations:** 1 MNJ Institute of Oncology and Regional Cancer Center (MNJIORCC), Hyderabad, Telangana India; 2 Department of Supportive Care, Princess Margaret Cancer Centre, University Health Network, Toronto, Canada; 3 Lien Centre for Palliative Care, Duke-NUS Medical School, Singapore, 169857 Singapore; 4 Saw Swee Hock School of Public Health, National University of Singapore, Singapore, Singapore

**Correction to: BMC Palliat Care**

10.1186/s12904-019-0465-y

Following publication of the original article [1], the corresponding author reported an error on the name of the fourth author. “Priya Chandran” should be “Priya Kumari”.

## References

[1] Jacob J, Palat G, Verghese N, Kumari P, Rapelli V, Kumari S, Malhotra C, Teo I, Finkelstein E, Ozdemir S (2019). Health-related quality of life and its socio-economic and cultural predictors among advanced cancer patients: evidence from the APPROACH cross-sectional survey in Hyderabad-India. BMC Palliat Care.

