# Supplementary material for: Health-related quality of life and its socio-economic and cultural predictors among advanced cancer patients: evidence from the APPROACH cross-sectional survey in Hyderabad-India
Source: BMC Palliat Care. 2019 Nov 5;18:94. doi: 10.1186/s12904-019-0465-y (PMC6833246; doi:10.1186/s12904-019-0465-y)
Supplement: Supplementary file 1 — Additional file 1. Survey instrument. [file 12904_2019_465_MOESM1_ESM.pdf]

SUPPLEMENTARY MATERIAL A

Asian Patient Perspectives Regarding Oncology Awareness, Care, and Health (APPROACH)

For interviewer: Fill in the following information after you have completed the questionnaire.

PARTICIPANT CODE: (FROM CONSENT FORM)

DATE OF INTERVIEW (DD/MM/YYYY): ___/___/_____

TIME STARTED: TIME ENDED: TOTAL INTERVIEW TIME:

INTERVIEWER NAME:

COUNTRY: INTERVIEW LANGUAGE:

NAME OF THE INSTITUTION:

PATIENT TYPE: ☐1 OUTPATIENT ☐2 INPATIENT

SITE OF RECRUITMENT:

☐1 DEPARTMENT OF MEDICAL ONCOLOGY

☐2 DEPARTMENT OF PALLIATIVE CARE

**Fill in the information from patient's medical records.**	PATIENT GENDER: ☐1 MALE ☐2 FEMALE
	PATIENT'S DATE OF BIRTH (DD/MM/YYYY):____/_____/_____
	TYPE OF CANCER: ______________________________

INTRODUCTION

We are conducting a survey to understand the quality of life of patients, quality of care they are currently receiving and their treatment preferences. Your opinions are important to the success of this study. The survey usually takes about **45 minutes**.
There are no right or wrong answers to the questions and you do not have to respond to any questions that you feel uncomfortable answering. Your identity and the information given will be kept strictly confidential and only group data will be reported.

SECTION S: SCREENER

S1 Have you ever been diagnosed with any of the following health conditions? Check all that apply.

	Health Conditions	Yes
☐1	Diabetes	☐
☐2	Heart conditions (e.g. heart attack, blocked blood vessels)	☐
☐3	Lung/Liver disease (e.g. bronchitis, hepatitis)	☐
☐4	Cancer	☐

[TERMINATE if option 4 'Cancer' is NOT checked]
[Thank you for your interest, but you are not eligible to continue with this survey]

[For questions A1 to A5, you do not need to read the response choices out loud to the patient. Allow the patient to first respond directly, and prompt him/her with relevant choices depending upon his/her response]

Refuse to answer

A1 What is your age?

☐ **years old** ☐ 888

A2 How many years of education have you completed (including higher education)?

☐ **years** ☐ 888

A3 What is your current marital status? ☐ 888

☐1	Married	☐4	Divorced
☐2	Separated	☐5	Never married
☐3	Widowed		

A4 What is your religion? ☐ 888

☐1	Hindu	☐7	Jewish
☐2	Muslim	☐8	Parsi/Zoroastrian
☐3	Christian (including Roman Catholic, Protestant, Orthodox, other)	☐9	Taoist
☐4	Sikh	☐10	Confucian
☐5	Buddhist	☐11	No religion
☐6	Jain	☐12	Other, specify: _______________

A4.1 What is your caste? [For India] ☐ 888

☐1	General	☐4	Other Backward Class (OBC)
☐2	Scheduled Caste (SC)	☐5	Don't know
☐3	Scheduled Tribe (ST)		

**A5**	**What type of cancer have you been diagnosed with?**				888
	☐1	Bladder	☐11	Lung	
	☐2	Brain	☐12	Nasopharyngeal	
	☐3	Breast	☐13	Oesophageal	
	☐4	Cervical	☐14	Ovarian	
	☐5	Colorectal	☐15	Oral	
	☐6	Endometrial	☐16	Pancreatic	
	☐7	Gastric	☐17	Prostate	
	☐8	Intestinal	☐18	Vulva	
	☐9	Kidney	☐19	Others, please specify ________	
	☐10	Liver	☐20	Don't know	

**A6**	**Do you know the current stage (i.e. severity) of your cancer?**		888
	☐1	Early Stage (Stage I, II or III)	
	☐2	Advanced Stage (Stage IV)	
	☐3	I don't know	
**A7**	**[If A6=2] What was the stage (i.e. severity) of your cancer when you <u>first</u> learned about it?**		888
	☐1	Early Stage (Stage I, II or III)	
	☐2	Advanced Stage (Stage IV)	
	☐3	I don't know	

SECTION B: QUALITY OF LIFE

B1 Below is a list of statements that other people with your illness have said are important or bothersome for them. Some of the items may appear to be repetitive, but please bear with us. Please indicate one number per line as it applies to the <u>past 7 days</u>.

		Not at all	A little bit	Some-what	Quite a bit	Very much	
B1.1 GP3	Because of my physical condition, I have trouble meeting the needs of my family	0	1	2	3	4	☐ 888
B1.2 GP5	I am bothered by side effects of treatment	0	1	2	3	4	☐ 888
B1.3 GP6	I feel ill	0	1	2	3	4	☐ 888
B1.4 GP7	I am forced to spend time in bed	0	1	2	3	4	☐ 888

		Not at all	A little bit	Some-what	Quite a bit	Very much	
B1.5 GS1	I feel close to my friends	0	1	2	3	4	☐ 888
B1.6 GS2	I get emotional support from my family	0	1	2	3	4	☐ 888
B1.7 GS3	I get support from my friends	0	1	2	3	4	☐ 888
B1.8 GS4	My family has accepted my illness	0	1	2	3	4	☐ 888
B1.9 GS5	I am satisfied with family communication about my illness	0	1	2	3	4	☐ 888
B1.10 GS6	I feel close to my partner (or the person who is my main support)	0	1	2	3	4	☐ 888
	Regardless of your current level of sexual activity, please answer the following question. If you prefer not to answer it, please mark this box ☐ and go to the next question.						
B1.11 GS7	I am satisfied with my sex life	0	1	2	3	4	

		Not at all	A little bit	Some-what	Quite a bit	Very much	
B1.12 GE1	I feel sad	0	1	2	3	4	☐ 888

		Not at all	A little bit	Some-what	Quite a bit	Very much	
B1.13 GE2	I am satisfied with how I am coping with my illness	0	1	2	3	4	888
B1.14 GE3	I am losing hope in the fight against my illness	0	1	2	3	4	888
B1.15 GE4	I feel nervous	0	1	2	3	4	888
B1.16 GE5	I worry about dying	0	1	2	3	4	888
B1.17 GE6	I worry that my condition will get worse	0	1	2	3	4	888

		Not at all	A little bit	Some-what	Quite a bit	Very much	
B1.18 GF1	I am able to work (include work at home)	0	1	2	3	4	888
B1.19 GF2	My work (include work at home) is fulfilling	0	1	2	3	4	888
B1.20 GF3	I am able to enjoy life	0	1	2	3	4	888
B1.21 GF4	I have accepted my illness	0	1	2	3	4	888
B1.22 GF5	I am sleeping well	0	1	2	3	4	888
B1.23 GF6	I am enjoying the things I usually do for fun	0	1	2	3	4	888
B1.24 GF7	I am content with the quality of my life right now	0	1	2	3	4	888

		Not at all	A little bit	Some-what	Quite a bit	Very much	
B1.25 Sp1	I feel peaceful	0	1	2	3	4	888
B1.26 Sp2	I have a reason for living	0	1	2	3	4	888
B1.27 Sp3	My life has been productive	0	1	2	3	4	888
B1.28 Sp4	I have trouble feeling peace of mind	0	1	2	3	4	888

B1.29 Sp5	I feel a sense of purpose in my life	0	1	2	3	4	888
		**Not at all**	**A little bit**	**Some-what**	**Quite a bit**	**Very much**	
B1.30 Sp6	I am able to reach down deep into myself for comfort	0	1	2	3	4	888
B1.31 Sp7	I feel a sense of harmony within myself	0	1	2	3	4	888
B1.32 Sp8	My life lacks meaning and purpose	0	1	2	3	4	888
B1.33 Sp9	I find comfort in my faith or spiritual beliefs	0	1	2	3	4	888
B1.34 Sp10	I find strength in my faith or spiritual beliefs	0	1	2	3	4	888
B1.35 Sp11NI	Difficult times have strengthened my faith or spiritual beliefs	0	1	2	3	4	888
B1.36 Sp12NI	Even during difficult times, I know that things will be okay	0	1	2	3	4	888
B1.37 Sp21	I feel hopeful	0	1	2	3	4	888

The next three questions will focus on your finances.

**B2**	**How well does the amount of money you have enable you to cover the cost of your treatment?**	888
	☐1	Very well
	☐2	Fairly well
	☐3	Poorly
**B3**	**How well does the amount of money you have take care of your daily needs?**	888
	☐1	Very well
	☐2	Fairly well
	☐3	Poorly
**B4**	**How well does the amount of money you have enable you to buy those little 'extras', that is, those small luxuries?**	888
	☐1	Very well
	☐2	Fairly well
	☐3	Poorly

Now, we would like to ask you some questions about your symptoms.

**B5**	**Throughout our lives, most of us have had pain from time to time (such as minor headaches, sprains, and toothaches). Have you had pain other than these everyday kinds of pain today?**	888
	☐1 Yes	
	☐2 No	
**B6**	**Please rate your pain by circling the one number that best describes your pain at its <u>worst</u> in the last 24 hours.**	888
	0 1 2 3 4 5 6 7 8 9 10<br>**No pain** … **Pain as bad as you can imagine**	
**B7**	**Please rate your pain by circling the one number that best describes your pain at its <u>least</u> in the last 24 hours.**	888
	0 1 2 3 4 5 6 7 8 9 10<br>**No pain** … **Pain as bad as you can imagine**	
**B8**	**Please rate your pain by circling the one number that best describes your pain on the <u>average</u>.**	888
	0 1 2 3 4 5 6 7 8 9 10<br>**No pain** … **Pain as bad as you can imagine**	

**B9**	**Please rate your pain by circling the one number that tells how much pain you have right now.**
	0 1 2 3 4 5 6 7 8 9 10<br>**No pain** … **Pain as bad as you can imagine**
**B10**	**What treatments or medications are you taking for your pain?**
**B10.1**	**In the last 24 hours, have you taken any medication for pain relief?**
	☐1 Yes
	☐2 No
**B11**	**(If B10.1 = 1) In the last 24 hours, how much relief have pain treatments or medications provided? Please circle the one percentage that most shows how much relief have you received.**<br>**No relief** 0% 10% 20% 30% 40% 50% 60% 70% 80% 90% 100% **Comple te relief**
**B12**	**(If B6–B9 ≠ 0 i.e. patient has experienced pain in last 24 hours) Please circle the one number that describes how, during the past 24 hours, pain has interfered with your:**
	**A. General activity**<br>**Does not interfere** 0 1 2 3 4 5 6 7 8 9 10 **Completely interferes**
	**B. Mood**<br>**Does not interfere** 0 1 2 3 4 5 6 7 8 9 10 **Completely interferes**
	**C. Walking ability**<br>**Does not interfere** 0 1 2 3 4 5 6 7 8 9 10 **Completely interferes**
	**D. Normal work (includes both work outside the home and housework)**<br>**Does not interfere** 0 1 2 3 4 5 6 7 8 9 10 **Completely interferes**
	**E. Relations with other people**<br>**Does not interfere** 0 1 2 3 4 5 6 7 8 9 10 **Completely interferes**

888

888

888

888

888

888

888

888

888

**F. Sleep**													888
**Does not interfere**	0	1	2	3	4	5	6	7	8	9	10	**Completely interferes**	
**G. Enjoyment of life**													888
**Does not interfere**	0	1	2	3	4	5	6	7	8	9	10	**Completely interferes**	

B13 Below is a list of symptoms that people with your illness commonly experience. Please circle or mark one number per line to indicate your response as it applies to the past 7 days.

	SYMPTOM MANAGEMENT	Not at all	A little bit	Some-what	Quite a bit	Very much	
B13.1 GP4	I have pain	0	1	2	3	4	888
B13.2 B1	I have been short of breath	0	1	2	3	4	888
B13.3 PAL5	I am constipated	0	1	2	3	4	888
B13.4 C2	I am losing weight	0	1	2	3	4	888
B13.5 O2	I have been vomiting	0	1	2	3	4	888
B13.6 PAL6	I have swelling in parts of my body	0	1	2	3	4	888
B13.7 PAL7	My mouth and throat are dry	0	1	2	3	4	888
B13.8 GP1	I have lack of energy	0	1	2	3	4	888
B13.9 GP2	I have nausea	0	1	2	3	4	888
B13.10	Any other symptom, please specify: ___________________		1	2	3	4	888

B14 (If any of B13.1 to B13.10 ≠0) Did you tell your doctor about your symptoms? 888

☐1	Yes
☐2	No

B15 (If B14=2) Why did you not tell your doctor about your symptoms? Check all that apply. 888

☐1	I can manage my symptoms myself

☐2	I want the doctor to focus on the treatment of my cancer
☐3	I did not get an opportunity to tell the doctor
☐4	I believe that it is easier to put up with symptoms than with the side effects that come from medicines to treat these symptoms.
☐5	I will use medicines only as a last resort to treat my symptoms
☐6	I believe that good patients avoid talking about symptom/s
☐7	I can get addicted easily to medicines needed to treat these symptoms
☐8	I believe that tolerating symptoms builds character—it's good for me.
☐9	Any other reason, specify____

B16 The following questions will help us to know how you are feeling. For each of the following questions, please indicate how you have been feeling in the PAST WEEK. You do not have to think too much to answer. Your immediate response is the best.

B16.1 I feel tense or wound up. ☐ 888

☐1	Most of the time
☐2	A lot of the time
☐3	From time to time, occasionally
☐4	Not at all

B16.2 I still enjoy the things I used to enjoy. ☐ 888

☐1	Definitely as much
☐2	Not quite so much
☐3	Only a little
☐4	Hardly at all

B16.3 I get a sort of frightened feeling as if something awful is about to happen. ☐ 888

☐1	Very definitely and quite badly
☐2	Yes, but not too badly
☐3	A little, but it doesn't worry me
☐4	Not at all

B16.4 I can laugh and see the funny side of things. ☐ 888

☐1	As much as I always could
☐2	Not quite so much now
☐3	Definitely not as much now
☐4	Not at all

**B16.5**	**Worrying thoughts go through my mind.**	
	☐1	A great deal of the time
	☐2	A lot of the time
	☐3	Not too often
	☐4	Very little
**B16.6**	**I feel cheerful.**	
	☐1	Never
	☐2	Not often
	☐3	Sometimes
	☐4	Most of the time
**B16.7**	**I can sit at ease and feel relaxed.**	
	☐1	Definitely
	☐2	Usually
	☐3	Not often
	☐4	Not at all
**B16.8**	**I feel as if I am slowed down.**	
	☐1	Nearly all the time
	☐2	Very often
	☐3	Sometimes
	☐4	Not at all

888

888

888

888

**B16.9**	**I get a sort of frightened feeling like ‘butterflies’ in the stomach.**	888
☐1	Not at all	
☐2	Occasionally	
☐3	Quite often	
☐4	Very often	
**B16.10**	**I have lost interest in my appearance.**	888
☐1	Definitely	
☐2	I don’t take as much care as I should	
☐3	I may not take quite as much care	
☐4	I take just as much care as ever	
**B16.11**	**I feel restless as if I have to be on the move.**	888
☐1	Very much indeed	
☐2	Quite a lot	
☐3	Not very much	
☐4	Not at all	
**B16.12**	**I look forward with enjoyment to things.**	888
☐1	As much as I ever did	
☐2	Rather less than I used to	
☐3	Definitely less than I used to	
☐4	Hardly at all	
**B16.13**	**I get sudden feelings of panic.**	888
☐1	Very often indeed	
☐2	Quite often	
☐3	Not very often	
☐4	Not at all	
**B16.14**	**I can enjoy a good book or radio or television program.**	888
☐1	Often	
☐2	Sometimes	
☐3	Not often	
☐4	Very seldom	

SECTION C: QUALITY OF CARE

C1 Now we would like to ask you some questions related to your experience in dealing with health care providers for your current illness. Think of all the healthcare providers (doctors, nurses, and allied health providers) that you have seen for your current illness since it was diagnosed. These include those in the hospital, polyclinics, private clinics, hospice, nursing homes or other places.

		Always (1)	Somet imes (2)	Never (3)	Not applicable (4)	
C1.1	How often did your doctors listen carefully to you?	☐	☐	☐		☐ 888
C1.2	How often did your doctors explain things in a way you could understand?	☐	☐	☐		☐ 888
C1.3	How often did your doctors give you as much information as you wanted about your treatments, including potential benefits and side effects?	☐	☐	☐		☐ 888
C1.4	How often did your doctors encourage you to ask all the cancer related questions you had?	☐	☐	☐		☐ 888
C1.5	How often did your doctor treat you with courtesy and respect?	☐	☐	☐		☐ 888
C1.6	How often were your nurses as helpful as you thought they should be?	☐	☐	☐		☐ 888
C1.7	How often did your nurses treat you with courtesy and respect?	☐	☐	☐		☐ 888
C1.8	How often did you think that your health problems related to your cancer or its treatment were handled quickly enough?	☐	☐	☐		☐ 888
C1.9	How often were you able to see the specialist doctors you wanted to see for your cancer?	☐	☐	☐	☐	☐ 888
C1.10	How often did the doctors, nurses, and other medical staff providing your care seem to work well together as a team?	☐	☐	☐		☐ 888

		Always (1)	Sometimes (2)	Never (3)	Not applicable (4)	
C1.11	How often did your doctors seem to be aware of treatments for your cancer that other doctors recommended?	☐	☐	☐	☐	888
C1.12	How often did you know who to ask when you had any questions related to your cancer or its treatments?	☐	☐	☐	☐	888
C1.13	How often did you feel that your doctors, nurses, and other medical staff did everything they could to treat your health problems related to your cancer or its treatments?	☐	☐	☐		888
C1.14	How often did you feel you were as much involved in decisions about your care as you wanted?	☐	☐	☐		888

C1.15 Overall how would you rate the quality of your health care since your diagnosis? 888

☐1	Excellent	☐4	Fair
☐2	Very good	☐5	Poor
☐3	Good		

C2 Now I would like you to think about your most recent visit again. I want to know your impressions of your most recent visit for health care. I would like you to rate your experiences using the following questions.

For your last visit to a health care provider, how would you rate the following:

		Very Good	Good	Moderate	Bad	Very Bad	
C2.1	...your experience of being treated respectfully?	1	2	3	4	5	888
C2.2	…how clearly health care providers explained things to you?	1	2	3	4	5	888
C2.3	…your experience of being involved in making decisions for your treatment?	1	2	3	4	5	888

<table>
<tr><td>C3</td><td colspan="6">I am now going to read you stories describing experiences that other people with your illness have had with health care services. I want you to think about these people's experiences as if they were your own. Once I have finished reading each story, I will ask you to rate what happened in the story as very good, good, moderate, bad or very bad.</td></tr>
<tr><td rowspan="3">C3.1</td><td colspan="6">Mrs. Gayatri went to a crowded clinic. No-one greeted her. She waited for 30 minutes when a nurse called for her for an examination behind a screen that separated the waiting area from the examination area.</td></tr>
<tr><td rowspan="2">How would you rate Mrs. Gayatri's experience of being greeted and talked to respectfully?...</td><td>Very Good</td><td>Good</td><td>Moderate</td><td>Bad</td><td>Very Bad</td></tr>
<tr><td>1</td><td>2</td><td>3</td><td>4</td><td>5</td></tr>
<tr><td rowspan="3">C3.2</td><td colspan="6">The doctor has very briefly explained to Mr. Mehta about his illness. He is very busy and there is a queue of patients waiting to see him. Mr. Mehta would like to know more about his illness, but feels that there is no time to ask questions. The doctor says goodbye to Mr. Mehta, and Mr. Mehta leaves the office.</td></tr>
<tr><td rowspan="2">How would you rate Mr. Mehta experience of how clearly health care providers explained things to him?</td><td>Very Good</td><td>Good</td><td>Moderate</td><td>Bad</td><td>Very Bad</td></tr>
<tr><td>1</td><td>2</td><td>3</td><td>4</td><td>5</td></tr>
<tr><td rowspan="3">C3.3</td><td colspan="6">The doctor ordered some blood tests and scan for Patel. Patel didn't know why he needed blood tests and scan and was worried until the doctor explained what they were for.</td></tr>
<tr><td rowspan="2">How would you rate Patel's experience of being involved in making decisions about his health care or treatment?</td><td>Very Good</td><td>Good</td><td>Moderate</td><td>Bad</td><td>Very Bad</td></tr>
<tr><td>1</td><td>2</td><td>3</td><td>4</td><td>5</td></tr>
</table>

888 Refuse to answer

888

888

SECTION D: PROGNOSIS, TREATMENT PREFERENCES, AND DECISION MAKING

D1 (If A6 ≠ 2) When did you first learn that you have cancer?

☐1	Less than 6 months ago	☐4	2 to 3 years ago
☐2	6 months to 1 year ago	☐5	More than 3 years ago
☐3	1 to 2 years ago		

888

D2 (If A6 ≠ 2) Who informed you about it?

☐1	Doctor	☐4	Guessed from the worsened condition
☐2	Family member	☐5	Other, please explain:________________
☐3	Learned by chance		

888

D3 (If A6 = 2) When did you first learn that you have advanced cancer?

☐1	Less than 6 months ago	☐4	2 to 3 years ago
☐2	6 months to 1 year ago	☐5	More than 3 years ago
☐3	1 to 2 years ago		

888

D4 (If A6 = 2) Who informed you about it?

☐1	Doctor	☐4	Guessed from the worsened condition
☐2	Family member	☐5	Other, please explain:________________
☐3	Learned by chance		

888

D5 How informed are you regarding how your illness will change over time?

☐1	Very informed	☐3	Unsure
☐2	Somewhat informed	☐4	Not informed

888

The next items ask what you think about the possible results of cancer treatments. Do you think …

D6 The current treatments you are taking for your cancer will help you live longer?

☐1	Yes
☐2	No
☐3	Not sure

888

**D7**	**The current treatments you are taking for your cancer will cure you?**	
	☐1	Yes
	☐2	No
	☐3	Not sure
**D8**	**There are other treatments (besides your current treatment) that will help you live longer?**	
	☐1	Yes
	☐2	No
	☐3	Not sure
**D9**	**There are other treatments (besides your current treatment) that will cure you?**	
	☐1	Yes
	☐2	No
	☐3	Not sure
**D10**	**Since diagnosis, who has been responsible for the most important decisions about your treatment? Check all that apply.**	
	☐1	Myself
	☐2	My family
	☐3	My doctors
**D11**	**(If more than one option is checked in D10.) Which statement best describes the role each person played when making decisions about your treatment?**	
	(If "myself" and "my family" are checked, options are)	
	☐1	I made the decisions after considering my family's opinion
	☐2	My family made the decisions after considering my opinion
	☐3	My family and I made the decisions together
	(If "myself" and "my doctors" are checked, options are)	
	☐4	I made the decisions after considering my doctors' opinions
	☐5	My doctors made the decisions after considering my opinion
	☐6	My doctors and I made the decisions together
	(If "my family" and "my doctors" are checked, options are)	
	☐7	My family made the decisions after considering my doctors' opinions
	☐8	My doctors made the decisions after considering my family's opinion
	☐9	My family and my doctors made the decisions together
	(If all three are checked, options are)	
	☐10	I made the decisions after considering my family's and doctors' opinions

888

888

888

888

888

	☐11	My family made the decisions after considering my and my doctors' opinions
	☐12	My doctors made the decisions after considering my and my family's opinions
	☐13	My family, my doctors and I made the decisions together
**D12**	**Since diagnosis, who do you wish had been responsible for the most important decisions about your treatment? Check all that apply.**	888
	☐1	Myself
	☐2	My family
	☐3	My doctors
**D13**	**(If more than one option is checked in D12.) Which statement best describes the role you wish each person had played when making decisions about your treatment?**	888
	(If "myself" and "my family" are checked, options are)	
	☐1	I wish that I made the decisions after considering my family's opinion
	☐2	I wish that my family made the decisions after considering my opinion
	☐3	I wish that my family and I made the decisions together
	(If "myself" and "my doctors" are checked, options are)	
	☐4	I wish that I made the decisions after considering my doctors' opinions
	☐5	I wish that my doctors made the decisions after considering my opinion
	☐6	I wish that my doctors and I made the decisions together
	(If "my family" and "my doctors" are checked, options are)	
	☐7	I wish that my family made the decisions after considering my doctors' opinions
	☐8	I wish that my doctors made the decisions after considering my family's opinion
	☐9	I wish that my family and my doctors made the decisions together
	(If all three are checked, options are)	
	☐10	I wish that I made the decisions after considering my family's and doctors' opinions
	☐11	I wish that my family made the decisions after considering my and my doctors' opinions
	☐12	I wish that my doctors made the decisions after considering my and my family's opinions
	☐13	I wish that I, my family and my doctors made the decisions together
**D14**	**If there is a difference in opinion between you and your family regarding the appropriate course of treatment for you, whose opinion do you think will be <u>most important</u> in deciding the treatment that you receive?**	
	☐1	My own

888

	☐2	My family’s
	☐3	My doctor’s

We will now ask you questions about your treatment preferences. ***The next questions that I am going to ask you are very sensitive in nature. Please skip the questions that you do not feel comfortable answering.***

D15 **If you had to make a choice now, would you prefer treatment that extends life as much as possible, or would you want treatment that gives you minimal pain and discomfort? Please choose a point in the scale below.** 888

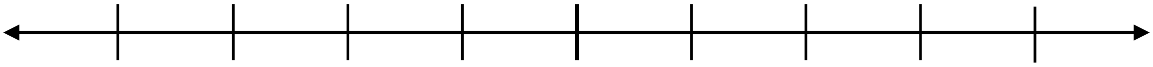

Extend life as much as possible<br>Severe pain or discomfort	Moderate life extension<br>Moderate pain or discomfort	No life extension<br>Minimal pain or discomfort

D16 **If you had to make a choice now, would you prefer treatment that extends life as much as possible, or would you want treatment that costs you less? Please choose a point in the scale below.** 888

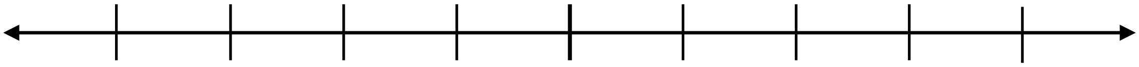

Extend life as much as possible<br>High cost	Moderate life extension<br>Moderate cost	No life extension<br>Less cost

D17 **Have you discussed your treatment and care preferences with your family member(s)?** 888

☐1	Yes	☐2	No

SECTION E: HOSPICE PALLIATIVE CARE AWARENESS AND UTILIZATION

E1 Please indicate if you are aware of hospice palliative care services? (*Hospice palliative care is a comprehensive program that focuses on providing relief of pain and other distressing symptoms, as well as ensuring emotional, spiritual and practical support for the patient and the family. These services can be given in the patient's home, in an in-patient hospice, hospice day care centre, in a palliative care clinic or in the ward of a hospital)*

☐1	Yes
☐2	No (Go to E8)
☐3	Not sure (Go to E8)

888

E2 How did you first learn about hospital palliative care services?

☐1	From doctors or other healthcare professionals	☐4	From the media
☐2	From family/friends	☐5	From others, please specify:________________
☐3	Through personal research		

888

E3 Did any doctor or other health care provider <u>recommend</u> hospice palliative care to you?

☐1	Yes
☐2	No (Go to E5)
☐3	Not sure (Go to E5)

888

E4 When did the doctor recommend hospice palliative care for you?

☐1	Within the last month	☐4	1 year ago
☐2	2 to 3 months ago	☐5	More than 1 year ago
☐3	4 to 6 months ago		

888

E5 Since the diagnosis of your illness, have you received hospice palliative care?

☐1	Yes
☐2	No (Go to E7)
☐3	Not sure (Go to E7)

888

E6 (If E5=1) Please tell us who had the FINAL say in deciding for you to receive hospice palliative care.

☐1	Me only	☐5	Me and my doctor
☐2	My family only	☐6	My family and my doctor
☐3	My doctor only	☐7	Me, my family and my doctor
☐4	Me and my family	☐8	Others, please specify:_________

888

**E7**	**[You do not need to read response choices out loud. Allow the patient to first respond directly, and prompt him/her with relevant choices depending upon his/her response]** **(If E5≠1) Why are you not receiving hospice palliative care at this time? Check all that apply.**	888
	☐1 I am still receiving treatment for my disease	
	☐2 I do not believe it's time for hospice palliative care	
	☐3 I do not think that hospice palliative care would be of help to me	
	☐4 Some of my doctors do not think that hospice palliative care would be of help to me	
	☐5 My family does not believe it's time for hospice palliative care	
	☐6 My family does not think that hospice palliative care would be of help to me	
	☐7 I don't know much about it	
	☐8 I think getting hospice palliative care will be like giving up on life	
	☐9 It is expensive	
	☐10 Any other reason, please specify:________________	
	The next questions that I am going to ask you are very sensitive in nature. We are asking you these questions to better understand your thoughts on your health, quality of life and the quality of care you receive from this hospital. Please skip the questions that you do not feel comfortable answering.	
**E8**	**Would you like to know how long you are likely to live under various treatment options?**	888
	☐1 No	
	☐2 Yes, in general terms (such as 'a few months' or 'a few years')	
	☐3 Yes, in specific terms (such as 'on average 6 months')	
	☐4 Not Sure	
**E9**	**[You do not need to read response choices out loud. Allow the patient to first respond directly, and prompt him/her with relevant choices depending upon his/her response]** **Considering your current health condition and treatment plan, how long do you think you are likely to live?**	888
	☐1 Less than 1 year	
	☐2 1 up to 2 years	
	☐3 2 up to 3 years	
	☐4 3 up to 5 years	
	☐5 5 up to 7 years	
	☐6 7 up to 10 years	
	☐7 More than 10 years, please specify ______________________________	

**E10**	**Some people have a strong preference concerning where they would like to spend their last days prior to death. If you had a chance, where would you like to be during the last days of your life?**	
	☐1	At my home
	☐2	In the home of a friend/family member
	☐3	In the hospital where I have been receiving medical care
	☐4	Hospice
	☐5	Nursing home
	☐6	Any other place, please specify:________________
	☐7	Doesn’t matter
**E11**	**Do you ever wish that your life would end sooner?**	
	☐1	Yes
	☐2	No
	☐3	Not sure

888

888

SECTION F: SELF BLAME AND SOCIAL STIGMA

F1 Have you smoked at least 100 times in your life?

☐ 1	Yes	**Answer Qs. F1.1 & F1.2**
☐ 2	No	**Skip to Qs. F2**
☐ 3	Don't know	

888

F1.1 How old were you when you started to smoke regularly?

☐ years old

888

F1.2 Are you currently smoking?

☐ 1	**Yes**
☐ 2	**No**

888

F2 Have you ever chewed betel nut/tobacco regularly?

☐ 1	Yes, I currently chew betel nut/tobacco regularly
☐ 2	Yes, I have chewed betel nut/tobacco in the past but not anymore
☐ 3	No

888

F2.1 (If F2= 1 or 2) How old were you when you started to chew betel nut/tobacco regularly?

☐ years old

888

F3 Before you were diagnosed with cancer, how often did you drink any type of alcoholic beverage?

☐ 1	Less than once a month
☐ 2	A few times a month
☐ 3	A few times a week
☐ 4	Daily

888

F3.1 (If F3≠1) Before you were diagnosed with cancer, how much alcohol did you usually have each time you had drinks?

☐ 1	1 – 2 drinks
☐ 2	3 – 4 drinks
☐ 3	5 or more drinks

888

F3.2 Are you currently consuming any alcoholic beverages?

☐1	Yes	☐2	No

888

F4 ***We have found that some people blame themselves for their cancer and some people don't blame themselves at all.***
How much do you blame yourself for:

		Not at all (1)	Somewhat (2)	Very much (3)	Completely (4)	
**i.**	Any behaviour that may have led to your cancer?	☐	☐	☐	☐	888
**ii.**	The kind of person you are (e.g., being the unlucky person who has things like cancer happen to them)?	☐	☐	☐	☐	888

F5 **To the extent of your knowledge, which of the following (if any) are reasons for the type of illness you have? Check all that apply.** 888

1	Smoking	☐
2	Chewing betel nut/tobacco	☐
3	Consumption of alcohol	☐
4	Being overweight	☐
5	Stress/ Anxiety	☐
6	Previous bad deeds	☐
7	God's will	☐
8	Old age	☐
9	Others, please specify _________________________	

F6 **We have found that some people feel differently about themselves and experience changes in their social interactions due to their cancer. Consider the statements below and indicate how often they happen for you.**

		Never (1)	Rarely (2)	Sometimes (3)	Often (4)	Always (5)	
**i.**	I feel others consider me responsible for my cancer.	☐	☐	☐	☐	☐	888
**ii.**	I am embarrassed when I tell people my diagnosis	☐	☐	☐	☐	☐	888
**iii.**	I feel ashamed for having developed cancer	☐	☐	☐	☐	☐	888
**iv.**	People avoid me because of my cancer	☐	☐	☐	☐	☐	888
**v.**	I have an urge to keep my cancer a secret	☐	☐	☐	☐	☐	888
**vi.**	I sense that others feel strained when they are around me.	☐	☐	☐	☐	☐	888

F7	**Do you think your family and friends think cancer is contagious?**			
	☐ 1	Yes, most of them	☐ 3	No
	☐ 2	Yes, some of them	☐ 4	Not sure

888

F8	**How much control do you believe you personally have over:**				
		**Absolutely no control (1)**	**Little bit of control (2)**	**Some control (3)**	**Total Control (4)**
**i.**	The stress from your illness?	☐	☐	☐	☐
**ii.**	Physical symptoms of your disease or side effects of your treatment (e.g., pain)?	☐	☐	☐	☐
**iii.**	The type of medical treatment you receive?	☐	☐	☐	☐
**iv.**	Whether your condition will get better?	☐	☐	☐	☐

888
888
888
888

F9	**Do you believe God will cure your illness one day?**	
	☐ 1	Yes
	☐ 2	No
	☐ 3	Not sure

888

F9.1	**[If F9=Yes] How will this happen?**	
	☐ 1	Through a miracle without medical treatment
	☐ 2	Through medical treatment

888

SECTION G: USE OF/INTEREST IN USING MENTAL HEALTH SERVICES

**G1**	**Have you seen any of the mental health care workers listed below as part of your cancer treatment? Check all that apply.**			888
	☐ 1	Psychiatrist	**Answer Qs. G2 to G4**	
	☐ 2	Psychologist		
	☐ 3	Medical social worker (for psychological support)		
	☐ 4	Community counsellor		
	☐ 5	Others, please specify ________________		
	☐ 6	Don't know	**Skip to Q. G5**	
	☐ 7	No		

**G2**	**[If G1= 1 to 5] What type of mental health service did you receive?**				888
	☐ 1	Medications	☐ 3	Support group	
	☐ 2	Therapy/counselling	☐ 4	Other, please specify:_______________	

**G3**	**[If G1= 1 to 5] Where did you receive mental health services?**		888
	☐1	At the hospital as part of inpatient treatment	
	☐2	At the hospital in an outpatient appointment	
	☐3	I found a mental health professional myself	
	☐4	As part of homecare service	

**G4**	**[If G1= 1 to 5] How helpful did you find receiving mental health services?**				888
	☐ 1	Very helpful	☐ 3	I am not sure	
	☐ 2	Quite helpful	☐ 4	Not helpful at all	

**G5**	**[If G1= 6 or 7] Would you use mental health services if you were referred?**		888
	☐ 1	Yes	
	☐ 2	No	
	☐ 3	Not sure	

**G6**	**Did you delay medical treatment for cancer for any reason?**			888
	☐ 1	Yes, I delayed my medical treatment for some time	**Answer Q. G6.1**	
	☐ 2	No, I sought medical treatment immediately	**Skip to Section H**	

G6.1	[If G6=1] What were the reasons for delay in your treatment? Check all that apply.		888
	☐1	Trouble in identifying a suitable treatment plan	
	☐2	Cost of treatment	
	☐3	Using alternative methods of healing before medical treatment (e.g., herbal treatment, prayer, homeopathy, diet therapy)	
	☐4	I felt overwhelmed/confused and did not know what to do	
	☐5	I felt ashamed to get help for my illness	
	☐6	My family did not think that I need medical treatment	
	☐7	Other reasons, please specify ________________________________	

SECTION H: USE OF COMPLEMENTARY AND ALTERNATIVE THERAPY

Complementary and Alternative Therapies refer to treatments that are used either (1) together with medical treatment, or (2) instead of medical treatment. Examples include herbal treatment, diet therapy, exercise (e.g., qi gong, yoga) and spiritual healing.

H1	**Since diagnosis of cancer, what type of complementary and alternative therapy have you used? Check all that apply.**			888
	☐1	Traditional Chinese Medicine		
	☐2	Traditional Indian Medicine (e.g., Ayurveda, unani, siddha)		
	☐3	Homeopathy		
	☐4	Western herbal/health supplements		
	☐5	Others, please specify ________________________________		
	☐6	None of above		
**H2**	**Have you used any of the therapies listed below for your cancer? Check all that apply.**			888
	☐1	Herbal medicine to consume	**Answer Qs. H2.1 to H2.6**	
	☐2	Diet therapy (e.g., macrobiotic diet)		
	☐3	Acupuncture/ acupressure/ moxibustion		
	☐4	Massage/ Reflexology		
	☐5	Exercise (e.g., qi gong, tai chi, yoga)		
	☐6	Spiritual/faith healing by others		
	☐7	Others, please describe ___________________		
	☐8	None of above	**Skip to next section**	
**H2.1**	**[If H2=1 to 7] When did you start using this therapy for cancer?**			888
	☐1	Before being treated in the hospital		
	☐2	While I was being treated in the hospital		

	☐3	After being treated in the hospital			
**H2.2**	**[If H2=1 to 7] Is the therapy meant to replace your medical treatment?**				888
	☐ 1	Yes	☐ 2	No	
**H2.3**	**[If H2=1 to 7] Did you tell your doctor that you are using this therapy for your cancer?**				888
	☐ 1	Yes			
	☐ 2	No			
**H2.3.1**	**[If H2.3=1] How supportive was your doctor of this therapy?**				888
	☐ 1	Supportive	☐ 3	Neutral	
	☐ 2	Not supportive	☐ 4	Don't know	
**H2.4**	**[If H2=1 to 7] What is the main reason for using this therapy?**				888
	☐1	To boost my immune system			
	☐2	To reduce side effects of treatment			
	☐3	To control my symptoms (e.g. pain). If so, describe the symptom ________________			
	☐4	To prolong life			
	☐5	To cure my illness			
	☐6	Others. Please describe ____			
**H2.5**	**[If H2=1 to 7] How long have you been using this therapy?**				888
	☐1	Less than 1 month			
	☐2	Between 1 to 3 months			
	☐3	Between 3 to 6 months			
	☐4	More than 6 months			
	☐5	I am no longer using this therapy			
**H2.6**	**[If H2=1 to 7] Roughly how much did you spend, in total, on complementary and alternative therapies in the last 3 months?** ___________________________				888

SECTION I: DEMOGRAPHICS

I1	**What is your current occupation?**				
	☐1	Farmer	☐6	Service- Government	
	☐2	Wage labourer	☐7	Homemaker	
	☐3	Skilled worker	☐8	Retired	
	☐4	Shop keeper	☐9	Unemployed	
	☐5	Self-employed	☐10	Others, please specify _________	
I2	**Were you working before you were seeking care at this hospital, for your illness?**				
	☐1	Working full-time	☐3	Retired and not working	
	☐2	Working part-time	☐4	Homemaker	
I3	**How many persons live in your household?**				
	☐	Number of people			
I4	**How would you rate the economic status of your household?**				
	☐1	Poor	☐3	Upper Middle Class	
	☐2	Lower Middle Class	☐4	Wealthy	

☐ 888

☐ 888

☐ 888

☐ 888

Thank you for completing the survey.

POST INTERVIEW: OBSERVATIONS OF THE INTERVIEWER

[Interviewer: Please fill in the following questions based on your observations during the interview.]

INT1 During the survey, was there someone either present in the room or in a room nearby who could hear the contents of the interview?

☐1	Yes, during most of the interview
☐2	Yes, during half of the interview
☐3	Yes, at times during the interview
☐4	For the most part, no other person was present to hear

INT2 To what extent did this person influence the patient's responses?

☐1	Would correct the patient's responses or prevent the patient from giving his or her own responses
☐2	Listened to the interview, but did not interrupt verbally
☐3	Hardly paid any attention to the interview
☐4	Didn't seem to have any effect on the patient's responses

INT3 The following concerns your impression of the patient.

		Yes (1)	Somewhat (2)	Not really (3)	No (4)	Not sure (5)
i.	Did you feel that the patient was mentally competent enough to provide adequate responses?	☐	☐	☐	☐	☐
ii.	Did you feel that the patient understood the questions?	☐	☐	☐	☐	☐
iii.	Did you feel that the patient was responsive to the interview?	☐	☐	☐	☐	☐
iv.	Did you feel the patient's hearing/visual difficulties adversely affected the survey?	☐	☐	☐	☐	☐

INT4 How tired did the patient appear after the interview?

☐1	Very
☐2	Somewhat
☐3	Not at all
